# Draft Genome Sequence of Bacillus velezensis BZR 277, a Prospective Biocontrol Agent against Phytoparasitic Nematodes

**DOI:** 10.1128/MRA.00266-21

**Published:** 2021-05-13

**Authors:** Anzhela M. Asaturova, Anna I. Нomyak, Alexander E. Kozitsyn, Margarita V. Shternshis, Andrey L. Rakitin, Alexey V. Beletsky, Andrey V. Mardanov, Nikolai V. Ravin

**Affiliations:** a Federal Research Center of Biological Plant Protection, Krasnodar, Russia; b Institute of Bioengineering, Research Center of Biotechnology, Russian Academy of Sciences, Moscow, Russia; University of Arizona

## Abstract

Bacillus velezensis strain BZR 277 is a rhizobacterium isolated from the rhizoplane of a winter oilseed rape plant from the Krasnodar region in Russia. This study presents the genome sequence of the rhizobacterium Bacillus velezensis BZR 277, which exhibited high antagonistic activity against the root-knot nematode Meloidogyne incognita Kof. The data on the genome sequence can help to identify pathways for the biosynthesis of metabolites responsible for nematicidal activity in order to improve crop protection against plant-parasitic nematodes.

## Abstract (precis)

Bacillus velezensis strain BZR 277 is a rhizobacterium isolated from the rhizoplane of a winter oilseed rape plant from the Krasnodar region in Russia. This study presents the genome sequence of the rhizobacterium Bacillus velezensis BZR 277, which exhibited high antagonistic activity against the root-knot nematode Meloidogyne incognita Kof.

## ANNOUNCEMENT

Meloidogyne incognita is a serious pest of vegetable crops. To control *M. incognita*, several species of *Bacillus* bacteria have been registered, including B. amyloliquefaciens (syn. B. velezensis), B. cereus, B. circulans, B. coagulans, B. firmus, B. licheniformis, B. megaterium, B. polymyxa, and B. subtilis (1). It is known that the synthesis of extracellular lytic enzymes and antibiotic compounds by *Bacillus* species can destroy the physiological integrity of nematode eggs and adults (2, 3).

The *B. velezensis* BZR 277 strain was isolated from the rhizoplane of winter oilseed rape plants at the experimental plots of the Bioresource Collection of the Federal State Budgetary Scientific Institution Federal Research Center for Biological Plant Protection (FSBSI FSCBPP) in Krasnodar, Russia. The strain was previously described as *B. amyloliquefaciens* BZR 277 based on its morphological features and analysis of the 16S rRNA gene sequence and was included in the departmental collection of useful microorganisms for agricultural purposes of the Russian Collection of Agricultural Microorganisms (accession number RCAM05296) as a producer of hydrolytic enzymes of the protease and lipase group and as an antagonist of the root-knot nematode Meloidogyne incognita Kof. (4, 5). Strain *B. velezensis* BZR 277 was registered as a promising biopreparation producer strain for the protection of vegetable, ornamental, and flower crops against root-knot nematode (6).

A liquid culture of the *B. velezensis* BZR 277 strain was obtained by cultivation in the original nutrient medium. The total DNA was isolated using the DNeasy PowerSoil kit (Qiagen). Genomic DNA was sequenced using Illumina and Oxford Nanopore platforms. The shotgun genome library was prepared using the NEBNext Ultra II DNA library prep kit (New England BioLabs, USA). The sequencing of this library on a MiSeq instrument (Illumina, San Diego, CA) generated 2,613,855 read pairs (2 × 300-nucleotide [nt] mode). In addition, genomic DNA was sequenced on a MinION device (Oxford Nanopore, UK) using the ligation sequencing kit 1D and FLO-MIN110 cells. Nanopore sequencing generated 79,498 reads with an average length of 5,881 nt. Paired overlapping MiSeq reads were merged using FLASH (7), and low-quality sequences were trimmed using Sickle v.1.33 (q = 30) (https://github.com/najoshi/sickle). Nanopore reads were *de novo* assembled using Flye v.2.7 (8). The consensus sequence was corrected with Pilon v.1.22 (9) using MiSeq reads.

As a result, the complete circular 3,913,640-bp-long genome with a G+C content of 46.6% was obtained; no plasmids were detected. Gene search and annotation were performed using the National Center for Biotechnology Information (NCBI) Prokaryotic Genome Annotation Pipeline (10). The default settings were used for all software. In the assembled genome, 9 16S-23S-5S rRNA gene clusters were found. Comparison of the 16S rRNA gene sequence of *B. velezensis* BZR 277 with the GenBank database showed that the strain belongs to the genus *Bacillus*. The strain was classified as Bacillus velezensis based on 97.6% average nucleotide sequence identity to Bacillus velezensis strain NRRL B-41580.

### Data availability.

The complete genome sequence of *B. velezensis* BZR 277 has been deposited in GenBank under the accession number CP064845. The version described in this paper is the first version (CP064845.1). The raw sequences have been deposited in the Sequence Read Archive under the accession numbers SRX9502288 and SRX9502287.
